# Development of individual competencies and team performance in interprofessional ward rounds: results of a study with multimodal observations at the Heidelberg Interprofessional Training Ward

**DOI:** 10.3389/fmed.2023.1241557

**Published:** 2023-09-27

**Authors:** Anika Mitzkat, Johanna Mink, Christine Arnold, Cornelia Mahler, André L. Mihaljevic, Andreas Möltner, Birgit Trierweiler-Hauke, Charlotte Ullrich, Michel Wensing, Jan Kiesewetter

**Affiliations:** ^1^Department of General Practice and Health Services Research, University Hospital Heidelberg, Heidelberg, Germany; ^2^Division of Neonatology, Department of Paediatrics, Inselspital, Bern University Hospital, University of Bern, Bern, Switzerland; ^3^Department of Nursing Science, University Hospital Tübingen, Tübingen, Germany; ^4^Department of General Visceral and Transplantation Surgery, University Hospital Ulm, Ulm, Germany; ^5^Department of Medical Examinations, Medical Faculty Heidelberg, Heidelberg, Germany; ^6^Department of General, Visceral and Transplantation Surgery, University Hospital Heidelberg, Heidelberg, Germany; ^7^Institute of Medical Education, LMU University Hospital, LMU München, München, Germany

**Keywords:** interprofessional education, interprofessional collaborative practice, interprofessional training ward, interprofessional ward rounds, evaluation, observation

## Abstract

**Introduction:**

Interprofessional training wards (IPTW) aim to improve undergraduates' interprofessional collaborative practice of care. Little is known about the effects of the different team tasks on IPTW as measured by external assessment. In Heidelberg, Germany, four nursing and four medical undergraduates (= one cohort) care for up to six patients undergoing general surgery during a four-week placement. They learn both professionally and interprofessionally, working largely on their own responsibility under the supervision of the medical and nursing learning facilitators. Interprofessional ward rounds are a central component of developing individual competencies and team performance. The aim of this study was to evaluate individual competencies and team performance shown in ward rounds.

**Methods:**

Observations took place in four cohorts of four nursing and four medical undergraduates each. Undergraduates in one cohort were divided into two teams, which rotated in morning and afternoon shifts. Team 1 was on morning shift during the first (t0) and third (t1) weeks of the IPTW placement, and Team 2 was on morning shift during the second (t0) and fourth (t1) weeks. Within each team, a tandem of one nursing and one medical undergraduate cared for a patient room with three patients. Ward round observations took place with each team and tandem at t0 and t1 using the IP-VITA instrument for individual competencies (16 items) and team performance (11 items). Four hypotheses were formulated for statistical testing with linear mixed models and correlations.

**Results:**

A total of 16 nursing and medical undergraduates each were included. There were significant changes in mean values between t0 and t1 in individual competencies (Hypothesis 1). They were statistically significant for all three sum scores: “Roles and Responsibilities”, Patient-Centeredness”, and “Leadership”. In terms of team performance (Hypothesis 2), there was a statistically significant change in mean values in the sum score “Roles and Responsibilities” and positive trends in the sum scores “Patient-Centeredness” and “Decision-Making/Collaborative Clinical Reasoning”. Analysis of differences in the development of individual competencies in the groups of nursing and medical undergraduates (Hypothesis 3) showed more significant differences in the mean values of the two groups in t0 than in t1. There were significant correlations between individual competencies and team performance at both t0 and t1 (Hypothesis 4).

**Discussion:**

The study has limitations due to the small sample and some sources of bias related to the external assessment by means of observation. Nevertheless, this study offers insights into interprofessional tasks on the IPTW from an external assessment. Results from quantitative and qualitative analysis of learners self-assessment are confirmed in terms of roles and responsibilities and patient-centeredness. It has been observed that medical undergraduates acquired and applied skills in collaborative clinic reasoning and decision-making, whereas nursing undergraduates acquired leadership skills. Within the study sample, only a small group of tandems remained constant over time. In team performance, the group of constant tandems tended to perform better than the group of random tandems. The aim of IPTW should be to prepare healthcare team members for the challenge of changing teams. Therefore, implications for IPTW implementation could be to develop learning support approaches that allow medical and nursing undergraduates to bring interprofessional competencies to team performance, independent of the tandem partner or team.

## 1. Introduction

Improving interprofessional collaborative practice (IPCP) has been formulated as a policy goal in healthcare worldwide (1), acknowledging the associations that have been found between IPCP, quality of care, and patient safety (2–5). Accordingly, in recent years, the topic of interprofessional care has also gained relevance in the educational policy of health professions (medicine, nursing, and other allied health professions) and is demanded as a curricular concept for these vocational training and study programs (1, 6–13). In Germany, interprofessional education (IPE) and interprofessional learning (IPL) have been implemented in the curricula at many sites (14, 15). In medicine, interprofessional competencies should be taught longitudinally, according to the new National Competency-Based Learning Objective Catalog of Undergraduate Medical Education (16). Interprofessional competencies are also explicitly described for nursing in the new vocational training regulations (17). Competencies should be acquired at the level of independent and situation-appropriate performance by the end of training or study. Interprofessional training wards (IPTW) are of particular importance for this level of competence, as they exhibit a high degree of complexity in direct patient care that enables learners to interact self-determinedly and self-responsibly to the greatest possible extent (18–20). IPTWs have been implemented at many hospitals worldwide (18, 21–36). IPTW addresses both profession-specific and interprofessional learning objectives, namely by having undergraduates from different healthcare professions (2–12 undergraduates, depending on the concept) take over the care of a certain number of patients as independently as possible under supervision by learning facilitators. Competency frameworks (37–40) are often used to formulate interprofessional learning objectives. The didactic concept builds on adult learning theories (41, 42), such as cognitive constructivism (43) and socio-constructivism (44). Interprofessional learning is also promoted through real-life placement (45, 46). Positive short-term effects of IPTW are described, especially with regard to a better understanding of professional roles, as well as the long-term effects of interprofessional competencies. Most studies on IPTW document learning outcomes based on students' self-reported evaluations (20). IPTW is also increasingly being implemented in Germany (47–49).

Together with the implementation of IPE/IPL in the curricula of health professions, there is an increasing need to evaluate it, especially with regard to its impact on the competencies for IPCP (50, 51). Questionnaires are often used for this purpose, which collect a structured self-assessment of the participant in IPE/IPL (52–54) and are mostly oriented toward competency frameworks (1, 39, 55). In Kirkpatrick's classification (56) of learning-related outcomes, as modified by Barr et al. (57), these studies primarily map knowledge and attitude-related changes (levels 1 and 2a/b). Studies that assess behavioral change (level 3) or impact on quality of care (level 4a/b) through third-party assessment are rare.

### 1.1. The Heidelberg interprofessional training ward

In 2017, an interprofessional training ward (Heidelberger Interprofessionelle Ausbildungsstation, HIPSTA) was implemented in an abdominal surgery ward at the university hospital in Heidelberg, Germany (46). Together with the IPTW in Mannheim and Freiburg, which started shortly thereafter, it was the first IPTW in Germany. At that time, the “ward within a ward” included two three-bed patient rooms and a dedicated ward office. Utilities were shared with the surrounding ward. Four students of human medicine (medical undergraduates, MU) in their practical year (the last year of a total of 6 years of study) and four nursing trainees (nursing undergraduates, NU) in their third year of training (the last year of a total of 3 years of vocational training) spent a 3- to 5-week placement on the HIPSTA, during which they were responsible for the patient as far as possible independently and on their own responsibility. The undergraduates work in two shifts on weekdays, with a 2-h overlap at noon. On weekends and at night, patients are cared for by the ward's regular nursing staff. The cohorts (4 NU + 4 MU) were assigned to early and late shifts in the weekly rotation. The respective teams of one shift (2 NU + 2 MU) were divided into two interprofessional tandems, which took over the care of the patients in one room each. One team of four participants was planned to start with the early shift for the whole first week; the other four were to start with the late shift. In the second week, the groups switch, and the participants who worked early shift in the first week work late shift in the second week, and vice versa. In weeks 3 and 4, they changed again, enabling each group to work in one shift for 5 days in a row and a weekly alternation of early and late shifts, resulting in 2 weeks of early and 2 weeks of late shifts for each participant in total.

As shown by a retrospective analysis of patient data (58), the patients to be cared for did not significantly differ from patients on the surrounding ward with regard to age, comorbidities, reason for admission, or data concerning surgery. The undergraduates were supervised by nursing and physician learning facilitators. A nursing facilitator was present throughout the morning shift. The physician facilitator was present for the morning ward rounds, midday handovers, and afternoon short rounds and was on call by phone the rest of the time. During the afternoon shift, an experienced nurse from the surrounding ward who had been well introduced to the HIPSTA concept was the contact person for the undergraduates. The learning facilitators interfered with patient care only when there was a concern that patient safety would otherwise be compromised. Otherwise, they remained in the background and only became active when requested by the undergraduates, answering questions or, in case participants asked for it, guided certain actions on the patient or in administration. They also provided feedback and fostered reflection and problem-solving processes. The daily routine at HIPSTA was structured by different practical learning phases in which the undergraduates learned both professionally and interprofessionally. On the morning shift, the interprofessional ward round took place. It started at approximately 8 a.m. A tandem of one NU and one MU conducted the round in the room they were caring for. The facilitators took part in the rounds but remained in the background. In addition, the nursing shift leader of the surrounding ward, a pharmacist, and other medical staff may also have been involved. The other tandem of the team also passively participated in the ward round. The round in the patient's room was usually preceded by a brief exchange outside the room on the current situation or on aspects that could not be discussed in front of the patient for certain reasons. After all patients in both rooms had been visited, a joint comprehensive debriefing of the information gained took place in the HIPSTA ward office. The further treatment, therapy, and care plan were developed jointly in tandem and coordinated with the nursing and physician facilitators.

For the overall evaluation of HIPSTA, a mixed-methods approach was chosen (59), which included quantitative and qualitative analyses. The results of the quantitative analyses of self-assessment questionnaires (60), the reconstructive analyses based on group interviews (61), and qualitative content analysis of personal interviews (62) show an acquisition of competence experienced by the learners with regard to collaboration, roles, responsibilities, and communication, more positive attitudes toward IPL and teamwork, and partial development of an (inter-)professional identity and socialization.

In addition to the self-reported assessment of the HIPSTA evaluation, behavioral change was captured via third-party observation. The interprofessional ward rounds were chosen as the observational setting because it was anticipated that observable interaction between the undergraduates and with the patients would show up particularly often. For this purpose, an instrument was developed (63), which is multimodal in design and assesses both individual competencies and team performance. By observing the ward rounds, it was intended to record whether and how individual competencies and team performance change over time by means of external assessment.

### 1.2. Aims and research questions

The aim of this article is to present and discuss the results of the structured ward round observation. Research questions were: How do nursing and medical undergraduates develop individual competences and team performance during their 4-week HIPSTA placement measured by external assessment in ward rounding? To what extent does the development of nursing and medical undergraduates differ? To what extent are individual competencies and team performance interdependent?

## 2. Methods

### 2.1. Design and data collection

**Figure 1:**
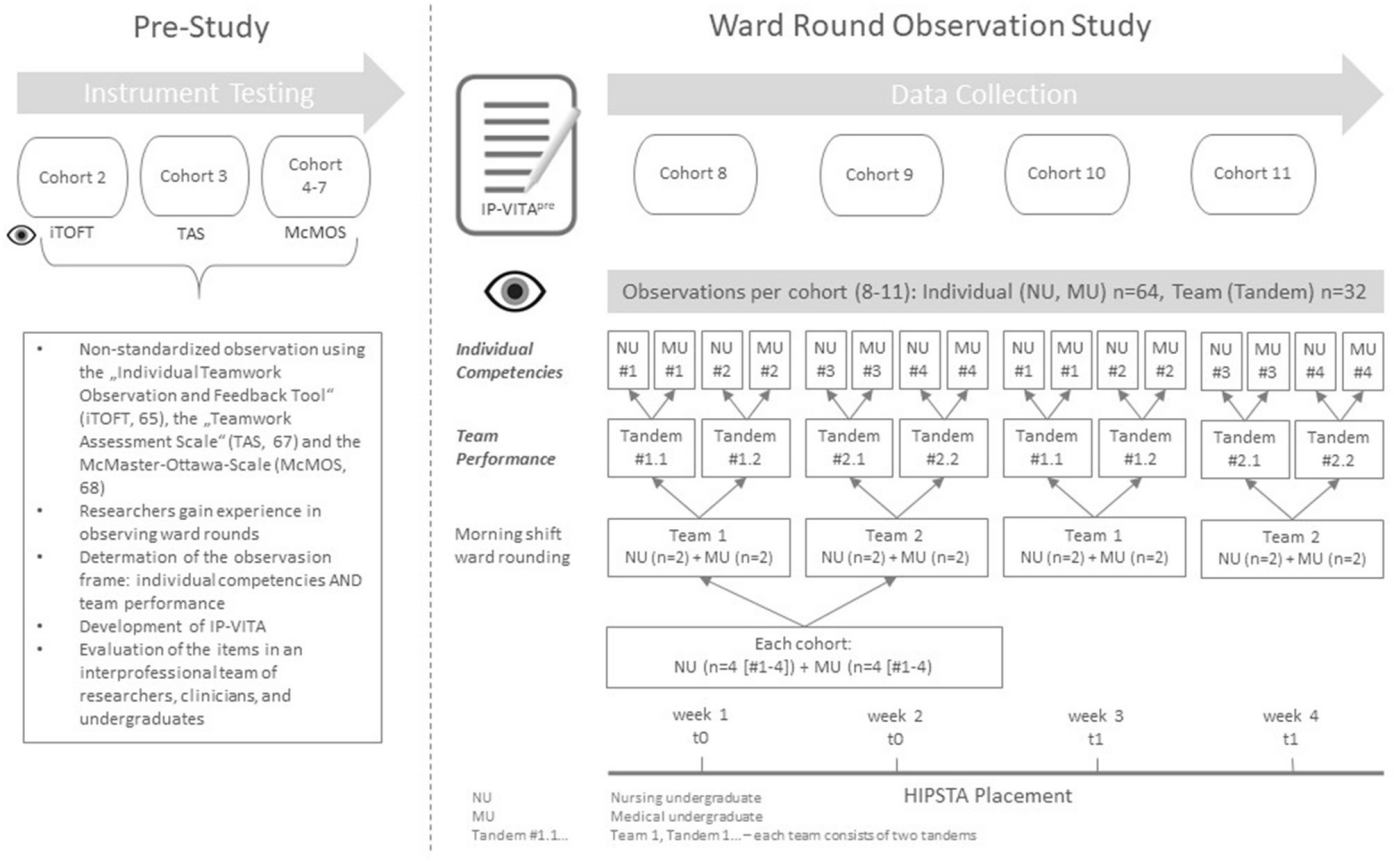
Study design.

Data were collected from January to May 2018 in four cohorts of HIPSTA in a pre- (beginning) post- (end) design. For this purpose, participants' rounds were observed in their first (t0) and last week (t1) of their morning shift, when ward rounds took place. Observation was conducted by two researchers each using the IP-VITA^pre^ [Individual competencies and team performance assessment tool (63)]. The instrument was developed empirically by first testing three instruments (64–66) available for the evaluation of interprofessional learning interventions with patient contact in a pre-study. The “Individual Teamwork Assessment Scale” (iTOFT) (64), the “Teamwork Assessment Scale” (TAS) (66), and the “McMaster-Ottawa Scale” (McMOS) (67) were used in at least one cohort (*n* = 4 observations). Afterwards, their use in the HIPSTA evaluation was discussed. It was decided that data should be collected at both the individual and team levels and that a separate instrument would be needed for this purpose. Therefore, an instrument, the IP-VITA, was developed from the experience made with the former instruments. As shown in Figure 1, the data presented were collected using the preliminary version of the instrument (IP-VITA^pre^). In this version, observable individual behavior was assessed by 16 items. Nine items were further developed from the instrument testing and adapted to the specific context of ward rounding. These items were evaluated on a 6-point Likert scale. Seven items compressed the CanMEDS model (68). Observable interaction within the tandems was assessed by 11 items on a 4-point Likert scale. Definitions (strongly agree/don't agree at all, to a very high degree/to a very low degree) were given only for the maximum expressions. Gradations were scored at intervals in relation to these two poles. Figure 1 gives an overview of the study design and data collection.

Each researcher observed one person in the tandem regarding individual competencies. Both took notes on the interaction and completed the team performance scale jointly after the observation in terms of intersubjective interpretation for each tandem.

### 2.2. Statistical analysis

All data analysis was performed in the IBM SPSS Statistics 22 software, except for the mixed model calculated for the team performance scale, which was performed in R. The dataset was cleaned by identifying outliers and extreme values and checking the dataset for plausibility. Missing values occurred when a skill or behavior was not observable. They were excluded on an item-by-item basis, as no systematic correlation between the missing values could be identified.

For statistical analysis, interval scaling was assumed for the Likert-scale data. Therefore, the mean and standard deviation are presented to describe the dataset.

Descriptive statistics for the sample were compiled. Welch *t*-test was calculated for the age difference in groups by profession (NU, MU).

The IP-VITA^pre^ was checked with regard to its internal consistency. Cronbach's alpha was calculated. Based on an exploratory factor analysis and theoretical considerations, three scores were formed for the individual competency scale and the team performance scale. For this purpose and for better comparability of graphical representation, six-point scaled items were converted to a four-point scale. Subscales were checked for internal consistency using Cronbach's alpha.

The following hypotheses were formulated for testing:

1. H1: values of individual competencies (item and score) differ in t0 and t1.
2. H1: values of team performance (item and score) differ in t0 and t1.
3. H1: there are differences in the mean values of the NU and MU groups at t0 and t1, and there are differences in the mean change over time.
4. H1: the values of individual competencies and team performance are correlated.

To describe the change in individual competencies and team performance over time and within the professional groups, a linear mixed model with restricted maximum likelihood (REML) and Satterthwaite's method with an F-test were calculated. For individual differences, the model included group (Hypothesis 3), time (Hypothesis 1), and their interaction as fixed, and participants as a random factor (Hypothesis 3). For team performance, the model included time as fixed and the NU/MU group as random factors (Hypothesis 2). Effects with *p* < 0.05 were considered significant. Trends that appeared to be particularly interesting were plotted graphically or described, even if they did not show a significant value.

Pearson correlations were performed to determine relationships between individual competencies and team performance (Hypothesis 4).

## 3. Results

### 3.1. Participant characteristics

Observations with the IP-VITA^pre^ took place in HIPSTA cohorts 8–11. A total of 16 nursing undergraduates (10 women and 6 men) and 16 medical undergraduates (3 women and 13 men) were included. No observation could be conducted with one medical undergraduate in cohort 10, and only one observation took place with one nursing undergraduate due to illness/shift change. In these cohorts, therefore, two other medical and one nursing undergraduate were observed at three instead of two data collection points. For individual competencies, the third observation was not included in the data analysis. At the team level, the observation was considered to be regular t0 or t1, as most of the other tandems also changed partners. Only four tandems remained constant across measurement time points t0 and t1, and the other 12 tandems worked with different partners at t1 than at t0.

**Figure 2:**
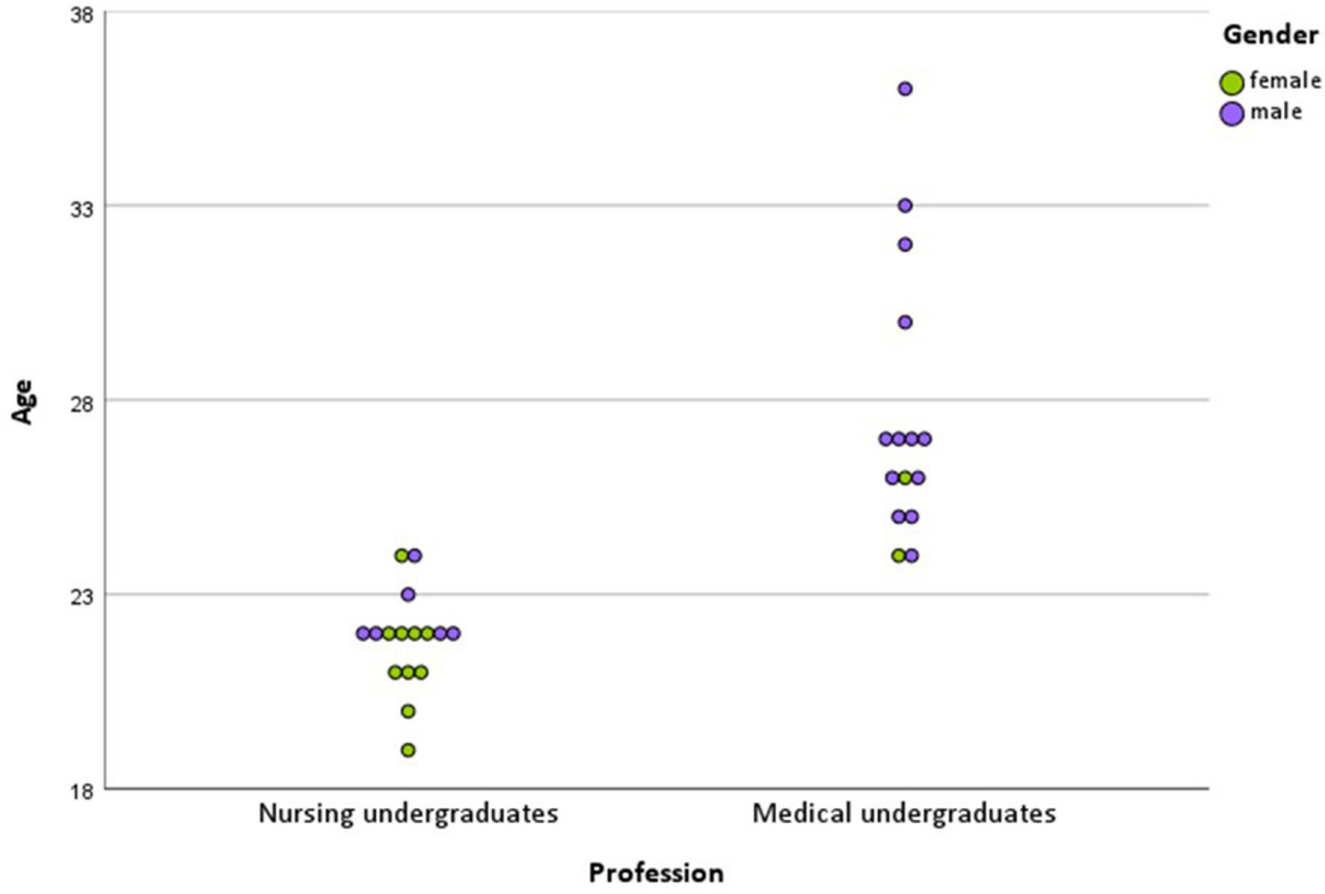
Dot plot of the study sample.

Table 1 and Figure 2 provide an overview of the included participants. The mean age was 21.8 ± 1 in the nursing group and 27.7 ± 3.4 in the medical group overall. This difference is significant (*p* < 0.001). There were significantly more women among nursing than among medical undergraduates (Fisher's test, *p* = 0.029). Based on this, the mean age difference is significantly different between male and female participants (*p* = 0.002).

**Table 1:** Study sample: profession, gender, and age.

**Profession**	**Gender**	***n***	**Age**	
			**x**	**SD**
Nursing	Female	10	21.40	1.35
	Male	6	22.50	0.83
Medical	Female	3	25.00	1.41
	Male	13	28.08	3.51
Total	Female	13	22.00	1.90
	Male	19	26.32	3.98

The number of patients in the two rooms varied according to surgery or overlapping times of discharge/admission. During 15 ward rounds at t0 and t1, each of the three patients had to be discussed. In 14 rounds at t1 and 13 rounds at t1, there had been two patients. In two rounds (t0 and t1), the rooms were occupied only by one patient. The patients' clinical appearance was heterogeneous and covered the whole spectrum of a general abdominal surgery ward. The nursing and physician learning facilitators were present in all except for two rounds, where once the physician and once the nurse were not present. The nursing ward manager was present in some rounds, a pharmacist in two, and an intern in two ward rounds.

### 3.2. Analysis of the IP-VITA^*pre*^

Exploratory factor analysis yielded a three-component solution for the individual scale and a four-component solution for the team performance scale. This was checked for plausibility in terms of content. Cronbach's alpha was calculated for all subscales. For the individual competencies, 14 of the 16 items could be combined in the three subscales “Roles and Responsibilities” (6 items, α = 0.891), “Patient-Centeredness” (3 items, α = 0.850), and “Leadership” (5 items, α = 0.845). The items “Active participation” and “CanMEDS Collaborator” were not included. The three subscales explain almost 70% of the variance in the data.

For the team performance scale, the subscales “Roles and Responsibilities” (2 items, α = 0.808), “Patient-Centeredness” (4 items, α = 0.844), and “Decision-Making/Collaborative Clinical Reasoning (CCR)” (3 items, α = 0.739) excelled. The fourth component was discarded due to a lack of content plausibility and low inner consistency. The items “Exchange between NU and MU present” and “Swift effective round” were not included. The team scores explained 70% of the total variance of the team performance scale.

### 3.3. Development of individual competencies

We hypothesized (Hypothesis 1) that there is a mean value change from t0 to t1. In the linear mixed model calculated for the influence of time (t0, t1), significant differences between moments in time showed up in all subscale scores, as can be seen in Table 2. The mean value of all except one item of the subscale “*Roles and Responsibilities*” increased from t0 to t1. The increase was highly significant in the item “defines clear goals for further treatment” (mean change 0.80, *p* = 0.001). This means, according to the descriptors of the item, that in the ward rounds observed at the end of the assignment on HIPSTA, the undergraduates more often explicitly addressed further treatment, prioritized actions, and named feasible goals, including their realization in time. The difference in time was also highly significant for the item “CanMEDS Manager” (mean change 0.74, *p* = 0.001). This means that the undergraduates were better organized and more effective with available resources in the second observed ward round. Overall, the increase in the subscale “*Roles and Responsibilities*” was significant (mean change 0.44, *p* = 0.016). The mean value of all items in the subscale “*Patient-Centeredness*” increased from t0 to t1. The increase was significant for the item “Discusses current patient information with patient involvement” (mean change 0.55, *p* = 0.019). This means that in the second observation, the undergraduates shared more information with each other and actively and clearly approached the patient to obtain or verify information. The increase in the item “CanMEDS Health Advocate” remained slightly below the significance threshold. No significant change could be observed regarding the handling of the patient's questions. The change in the subscale “*Patient-Centeredness*” over time is at the significance threshold (mean change 0.40, *p* = 0.049). The differences in means over time are significant in the subscale “*Leadership*” (mean change 0.39, *p* = 0.015). At the item level, the mean change of “Self-confident/sovereign appearance” (mean change 0.052, *p* = 0.032) and “CanMEDS Professional” (mean change 0.32, *p* = 0.030) was significant. This means that the undergraduates were more confident, including in terms of verbal expression, and gave the impression of being confident about the process of ward rounding. The mean chance over time in the item “CanMEDS Expertise” was highly significant (mean change 0.52, *p* = 0.001). This means that a higher level of diagnostic and therapeutic skills could be observed in t1. For the items that were not listed in a subscale, a significant difference in the mean value for the item “Active participation” could be shown (mean change 0.55, *p* = 0.002). This means that the undergraduates showed up more proactive and less reactive. The change over time in the item “CanMEDS Collaborator” was not significant.

**Table 2:** Development of individual competencies.

	**t0**		**t1**		
	***n***	**Mean (SD)**	***n***	**Mean (SD)**	**p** ^*^
**Roles and responsibilities (Score RR)**	31	**2.22** (0.720)	30	**2.66** (0.704)	**0.016**
Defines clear goals for further treatment (Defines goals)	30	**2.26** (0.882)	29	**3.06** (0.943)	**0.001**
Takes over tasks	27	2.71 (0.966)	27	3.17 (0.928)	0.074
Distributes tasks	22	1.70 (0.820)	27	1.80 (0.815)	0.606
Recognizes own knowledge gaps and asks questions (Recognizes knowledge gaps)	27	2.33 (1.043)	28	2.71 (0.989)	0.270
CanMEDS Scholar	29	2.38 (0.820)	26	2.50 (0.707)	0.547
CanMEDS Manager	31	**2.06** (0.814)	30	**2.80** (0.925)	**0.001**
**Patient-Centeredness (Score PC)**	31	**2.36** (0.727)	30	**2.76** (0.907)	**0.049**
Discusses current patient information with patient involvement (Patient involvement)	31	**2.51** (0.910)	30	**3.06** (1.017)	**0.019**
Ensures that the patients' questions are asked and answered (Patient questions)	30	2.36 (0.862)	26	2.63 (1.132)	0.426
CanMEDS health Advocate	30	2.20 (0.664)	28	2.64 (0.989)	0.052
**Leadership (Score LS)**	31	**2.54** (0.584)	30	**2.93** (0.654)	**0.015**
Ensures that all team members receive all information (Team info)	31	2.68 (0.882)	29	3.06 (0.826)	0.063
Self-confident/sovereign appearance	31	**2.68** (0.882)	30	**3.20** (0.896)	**0.032**
CanMEDS Communicator	31	2.68 (0.653)	30	2.93 (0.828)	0.163
CanMEDS Professional	31	**2.48** (0.626)	30	**2.80** (0.664)	**0.030**
CanMEDS Expertise	27	**2.22** (0.506)	27	**2.74** (0.526)	**0.001**
**Not included in subscales**					
Active participation	31	**3.03** (0.925)	30	**3.58** (0.689)	**0.002**
CanMEDS Collaborator	31	2.68 (0.702)	30	2.77 (0.898)	0.676

^*^p-value for F-test. The bold values indicate significant at *p* < 0.05.

### 3.4. Development of team performance

We hypothesized (Hypothesis 2) that the mean values of the team performance scale (item and score) differ in t0 and t1. A linear mixed model was calculated that considered that most of the tandems were not constant from t0 to t1 of the observation of team performance, which means that different nursing and medical undergraduates performed the ward round, respectively. As can be seen in Table 3, there was a non-significant negative trend in the difference of mean scores from t0 to t1 in the items “Patient questions are answered” and “Swift effective rounds”. For all other items, there was a positive trend, but it was also mostly non-significant. The significant change in the mean sum scores of “Roles and Responsibilities” (mean change 0.67, *p* = 0.008) is accounted for by the highly significant difference t0 to t1 in the item “Roles are clearly assigned” (mean change 0.75, *p* = 0.002). This means that the undergraduates showed a higher level of role awareness in the second observed ward round, behaved more according to their own professional roles, and seemed to acknowledge the role of the other profession.

**Table 3:** Development of team performance.

	**t0**		**t1**		
	***n***	**Mean (SD)**	***n***	**Mean (SD)**	**p** ^*^
**Roles and responsibilities (Core RR)**	16	**2.37** (0.562)	16	**3.00** (0.948)	**0.008**
Responsibilities are clarified	11	2.27 (0.786)	11	2.36 (1.027)	0.232
Roles are clearly assigned	16	**2.5** (0.516)	16	**3.25** (1.000)	**0.002**
**Patient-centeredness (Score PC)**	16	2.20 (0.647)	16	2.44 (0.807)	0.417
Patient is involved in information collection (Patient info)	16	2.75 (0.856)	16	3.13 (0.957	0.159
Patient is involved in the decision-making process (Patient CCR)	15	1.73 (0.799)	15	2.07 (0.884)	0.251
Patient questions are answered (Patient questions)	14	2.93 (0.616)	14	2.79 (1.122)	0.650
Goals are defined with the patient (Patient goals)	16	1.56 (0.727)	16	1.81 (0.911)	0.641
**Decision-making/CCR (Score CCR)**	16	2.58 (0.430)	16	3.02 (0.811)	0.059
Relevant nursing information is present (Nursing info)	16	2.5 (0.516)	16	2.88 (1.204)	0.118
Relevant medical information is present (Medical info)	16	2.69 (0.479)	16	3.19 (0.911)	0.092
Further procedure is planned by the team (Team planning)	13	2.46 (0.776)	13	3.00 (0.577)	0.212
**Not included in subscales**					
Exchange between NU and MU is present (NU MU exchange)	16	2.75 (1.125)	16	2.81 (0.911)	0.761
Swift effective round (Swift round)	16	3.06 (0.680)	16	3.00 (0.632)	0.724

^*^p-value for F-test.

**Figure 3:**
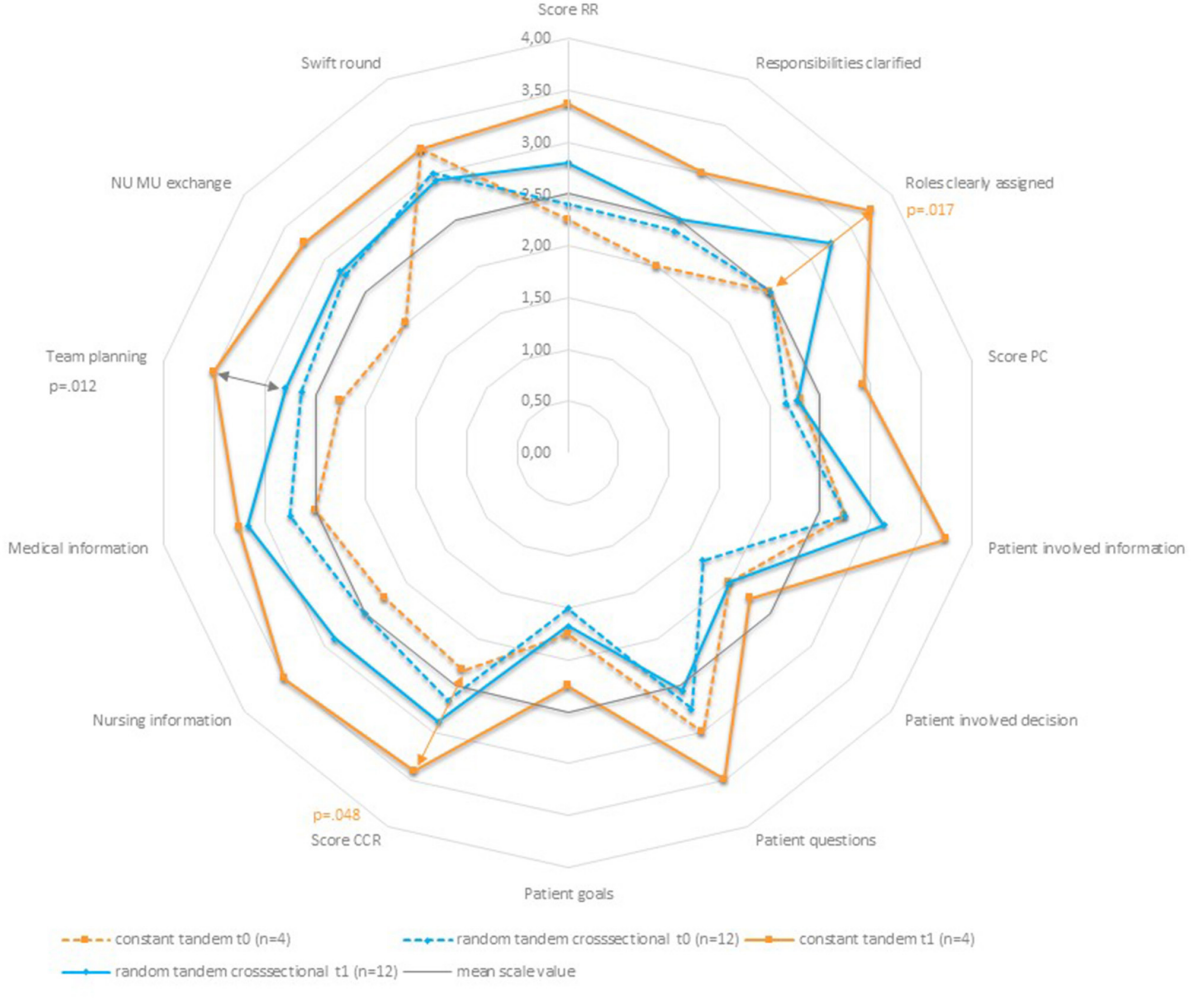
Team performance-spider chart of mean values.

It is important to emphasize that most of the tandems (*n* = 12) at t1 were not composed identically to the observation at t0. The analysis of the development in the overall sample describes that two random individuals interacted at the beginning and end of their assignment in a ward round. Thus, the tandem is more of a theoretical construct than an empirical one, since individuals (NU and MU) in the tandems did not remain constant as would have been intended in the study design (Figure 1). In the following, therefore, the mean change over time is presented only for the small group of tandems (*n* = 4) that were identical at t0 and t1. No p-values are given for the change in means over time for the group of random tandems. Rather, these data can be viewed as a cross-sectional investigation with random tandems at t0 and t1, respectively, with which the group of constant tandems (seen as cross-sectional) is compared in Figure 3 and Table 4. Within the group of constant tandems, all items and scores except for the item “Swift effective round”, which remained the same from t0 to t1 (mean change 0.00, *p* = 1), showed a clear positive tendency. However, this trend is significant only for the item “Roles are clearly assigned” (mean change 1.00, *p* = 0.017) and the score “*Decision-making/CCR*” (mean change 1.00, *p* = 0.48). Compared with all other tandems in t1, the team performance of the constant tandems (CT) was better across all items and scores than in the group of random tandems (RT). An exception is the item “swift effective round” (CT mean 3.00 ± 0.916, RT mean 3.00 ± 0.603, *P* = 1), which was identical. However, the differences between constant and random tandems are significant only for the item “Further procedure is panned by the team” (CT mean 3.67 ± 0.577, RT mean 2.82 ± 0.405, *p* = 0.012).

**Table 4:** Development of team performance in constant tandems.

	**Constant tandems**					**t1 compared with random tandem cross-sectional**		
	**t0**		**t1**		**t0** > **t1**	**Random tandem t1**		
	***n***	**Mean (SD)**	***n***	**Mean (SD)**	**p** ^*^	***n***	**Mean (SD)**	**p** ^*^
**Roles and responsibilities**	4	2.25 (0.645	4	3.12 (0.629)	0.063	12	2.95 (1.054)	0.772
Responsibilities are clarified	4	2.00 (0.816)	4	2.67 (0.577)	0.207	12	2.64 (1.120)	0.968
Roles are clearly assigned	4	**2.50** (0.577)	4	**3.50** (0.577)	0.**017**	12	3.17 (1.115)	0.582
**Patient-Centeredness**	4	2.31 (0.661)	4	2.68 (1.179)	0.248	12	2.36 (0.693)	0.593
Patient is involved in information collection	4	2.75 (0.947)	4	3,25 (1.500)	0.114	12	3.08 (0.793)	0.774
Patient is involved in the decision-making process	4	2.00 (1.000)	4	2.25 (0.957)	0.751	12	2.00 (0.853)	0.629
Patient questions are answered	4	3.00 (0.000)	4	3.00 (1.414)	0.203	12	2.73 (1.009)	0.682
Goals are defined with the patient	4	1.75 (0.500)	4	2.25 (0.957)	0.390	12	1.67 (0.888)	0.282
**Decision-making/CCR**	4	**2.33** (0.471)	4	**3.33** (0.902)	**0.048**		2.91 (0.792)	0.392
Relevant nursing information is present	4	2.25 (0.500)	4	3.50 (1.000)	0.067	12	2.67 (1.231)	0.243
Relevant medical information is present	4	2.50 (0.577)	4	3.25 (0.957)	0.228	12	3.17 (937)	0.880
Further procedures are planned by the team	4	2.25 (0.957)	4	**3.67** (0.577)	0.067	11	**2.82** (0.405)	**0.012**
**Not included in subscales**								
Exchange between NU and MU is present	4	2.00 (0.816)	4	3,25 (0.957)	0.094	12	2.67 (0.888)	0.282
Swift effective round	4	3.25 (0.500)	4	3.00 (0.916)	1.000	12	3.00 (0.603)	1.000

^*^p-value for Satterthwaite's F-test. The bold values indicate significant at *p* < 0.05.

### 3.5. Differences between groups by profession

We hypothesized (Hypothesis 3) that there are group differences in the mean values of NU and MU in t0 and t1 and in the mean change over time. The linear mixed model was calculated to check for the subscales in each case: whether the groups of nursing undergraduates (NU group) and medical undergraduates (MU group) differed at time points t0 and t1, whether there was a change from t0 to t1 in each group itself, and whether these changes differed between groups. With regard to the two items not listed in the subscales, the item “Active participation” showed a highly significant group difference at t0 (NU mean 2.5 ± 0.903, MU mean 3.6 ± 0.529, *p* < 0.001), which leveled off somewhat at t1 but remained significant (NU mean 3.3 ± 0.844, MU mean 3.84 ± 0.356 *p* = 0.036). The difference over time t0 to t1 was highly significant for the NU group (mean change 0.8, *p* = 0.008) but not for the MU group (mean change 0.24, *p* = 0.162). However, the difference in trend was not significant (0.078) which means, that both groups developed to a similar extent, albeit at a different level. There was no significant group difference for the item “CanMEDS Collaborator” in either t0 (NU mean 2.56 ± 0.814, MU mean 2.80 ± 0.561, *p* = 0.355) or t1 (NU mean 2,73 ± 0.961, MU mean 2.80 ± 0.862, *p* = 0.843). None of the groups had a significant change over time (NU mean change 0.17, *p* = 0.597, MU mean change 0.00, *p* = 1). There was also no difference in the range of development over time (*p* = 0.676).

#### 3.5.1. Roles and responsibilities

**Figure 4:**
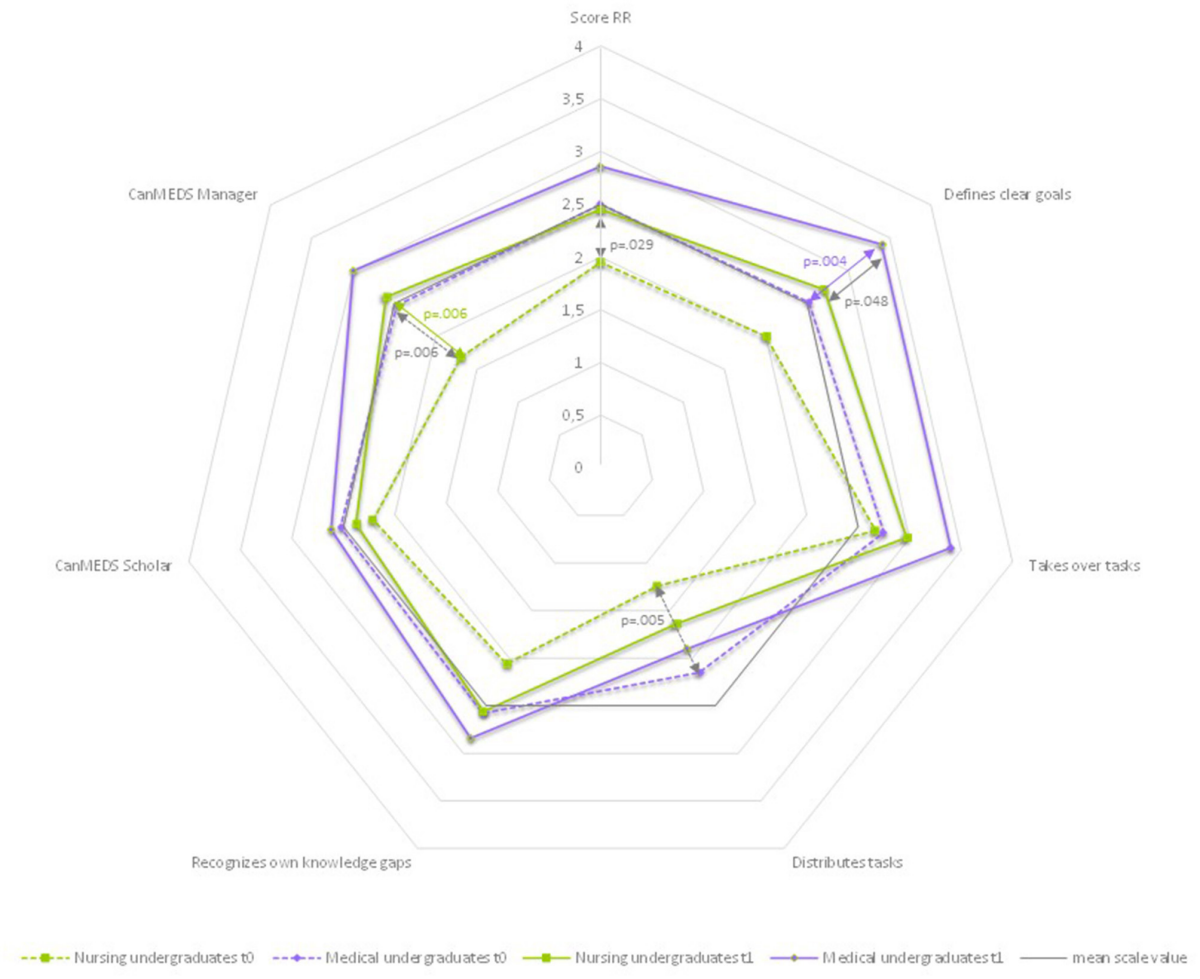
Differences by profession: *roles and responsibilities*-spider chart of mean values.

As shown in Table 5 and Figure 4, at t0, the MU group has higher mean values on all items than the NU group. This is highly significant for the items “Distributes tasks” (NU mean 1.24 ± 0.419, MU mean 2.15 ± 0.827, *p* = 0.005) and “CanMEDS Manager” (NU mean 1.69 ± 0.793, MU mean 2.47 ± 0.649, *p* = 0.006). This means that medical undergraduates asked nursing undergraduates more often to complete specific tasks later in the day and also exchanged about the timing of completion than the other way around. And for the “CanMEDS Manager”, which was also significant in the total sample, they showed more behaviors that served the effective organization of the ward routine. Accordingly, the mean value of the sum score “*Roles and Responsibilities*” is significantly higher than in the NU group. Interestingly, none of these differences are still significant in t1. Instead, the difference in means between the groups NU and MU in the item “Defines clear goals” is significant in t1 (NU mean 2.71 ± 1.125, MU mean 3.40 ± 0.600, *p* = 0.46). This means that the medical undergraduates more often showed behaviors that served to prioritize the further course of treatment and more often named explicit feasible goals, including their implementation in terms of time. Looking at the development from t0 to t1 in the respective groups, we find that the difference in terms of time is highly significant in the MU group (mean change 0.004, *p* = 0.004) but not in the NU group. But as seen in the data, the NU group also developed to a relatively high degree with respect to goal setting, although not at the 0.05 significance level. However, the differences in group development overall are not significant. Both groups developed similarly in all items and scores, albeit with different initial mean values. While the MU group evolved primarily in terms of treatment goal setting, the change over time in the NU group in the “CanMEDS Manager” item was highly significant (mean change 0.91, *p* = 0.006). We observed that the nursing undergraduates in t1 took more responsibility for the effective organization of ward procedures. Another finding is that while in t0 the differences in the mean values of the MU group compared to those of the NU group in the item “Distributes tasks” were highly significant, they are no longer so in t1. Instead, there is a significant change over time in the MU group in the item “Takes over tasks”. This means that the medical undergraduates observably expressed more frequently which tasks they would complete in the further course of the day.

**Table 5:** Differences by profession – score “*Roles and responsibility*.”

	**t0**					**t1**					**Development**		
	**NU**		**MU**			**NU**		**MU**			**NU t0** > **t1**	**MU t0** > **t1**	**group diff** ^*^
	***n***	**Mean (SD)**	***n***	**Mean (SD)**	**p** ^**^	***n***	**Mean (SD)**	***n***	**Mean (SD)**	**p** ^**^	**p** ^**^	**p** ^**^	**p** ^**^
**Score RR**	16	**1.95** (0.685)	15	**2.50** (0.662)	**0.029**	15	2.45 (0.826)	15	2.86 (0.503)	0.107	0.076	0.104	0.690
Defines clear goals	15	2.00 (0.807)	15	2.52 (0.903)	0.108	14	**2.71** (1.125)	15	**3.40** (0.600)	**0.048**	0.059	**0.004**	0.718
Takes over tasks	14	2.67 (1.057)	13	2.75 (0.898)	0.830	14	2.97 (1.139)	13	3.40 (0.600)	0.238	0.477	**0.041**	0.508
Distributes tasks	10	**1.24** (0.419)	12	**2.15** (0.827)	**0.005**	13	1.64 (0.792)	14	1.90 (0.872)	0.355	0.158	0.646	0.179
Recognizes own knowledge gaps	13	2.06 (0.921)	14	2.58 (1.119)	0.198	13	2.56 (1.162)	15	2.84 (0.832)	0.481	0.229	0.491	0.646
CanMEDS Scholar	14	2.21 (0.802)	15	2.53 (0.834)	0.304	13	2.38 (0.768)	13	2.62 (0.650)	0.417	0.579	0.776	0.833
CanMEDS Manager	16	**1.69** (0.793)	15	**2.47** (0.640)	**0.006**	15	2.60 (910)	15	3.00 (0.926)	0.243	**0.006**	0.077	0.373

^*^Difference in development from t0 to t1 between groups. ^**^p-value for Satterthwaite's F-test. The bold values indicate significant at *p* < 0.05.

#### 3.5.2. Patient-centeredness

**Figure 5:**
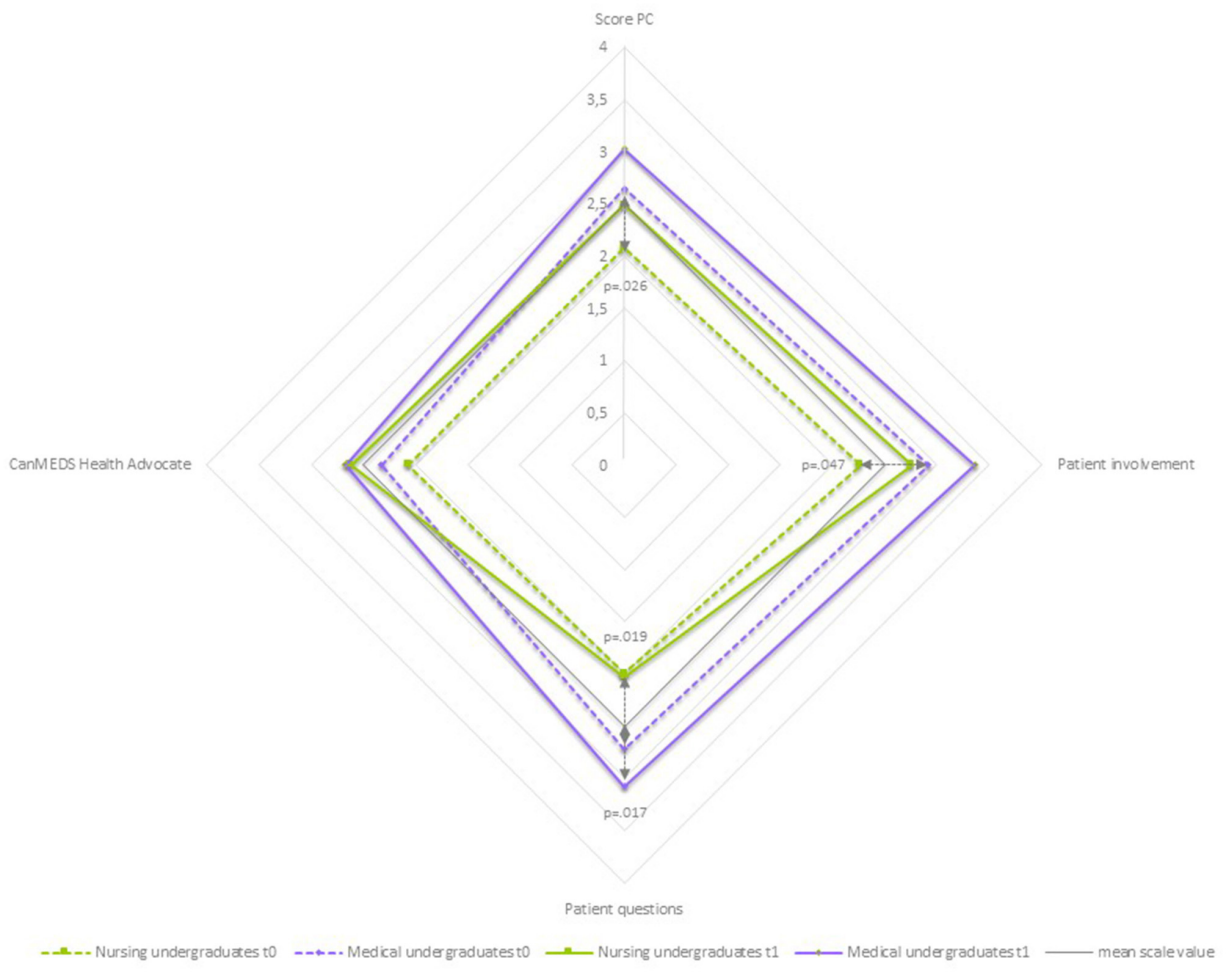
Differences by Profession: *patient-centeredness*-spider chart of mean values.

As shown in Table 6 and Figure 5, there were also differences in the mean values of the NU group compared to the MU group in the scale “*Patient-Centeredness*” at t0. Interestingly, all the mean values of the MU group are higher than those of the NU group. The difference is significant for the items “Discusses current patient information with patient involvement” (NU mean 2.27 ± 0.951, MU mean 2.92 ± 0.758, *p* = 0.47), “Ensures that the patients' questions are asked and answered” (NU mean 2.00 ± 0.740, MU mean 2.72 ± 0.844, *p* = 0.019), and the sum score (NU mean 2.08 ± 0.683, MU mean 2.65 ± 0.672, *p* = 0.026). This means that for the medical undergraduates, it was observed more frequently that they actively approached the patient to obtain and verify information, responded to the patient's questions, and included the patient in the goal-setting process for further treatment. In t1, the difference in mean values between the groups is still significant with regard to the item “Patients question” (NU mean 2.03 ± 0.1007, MU mean 3.08 ± 0.035, *p* = 0.017). In both groups, there is a positive trend in the mean values of t0 compared with t1. However, this was not significant for any of the groups. The two groups of NU and MU develop similarly in all items and the score, with different initial mean values.

**Table 6:** Differences by profession – score “*Patient-centeredness*.”

	**t0**					**t1**					**Development**		
	**NU**		**MU**			**NU**		**MU**			**NU t0** > **t1**	**MU t0** > **t1**	**group diff** ^*^
	***n***	**Mean (SD)**	***n***	**Mean (SD)**	**p** ^**^	***n***	**Mean (SD)**	***n***	**Mean (SD)**	**p** ^**^	**p** ^**^	**p** ^**^	**p** ^**^
**Score PC**	16	**2.08** (0.683)	15	**2.65** (0.672)	**0.026**	15	2.49 (0.939)	15	3.03 (0.816)	0.104	0.175	0.177	0.929
Patient involvement	16	**2.27** (0.951)	15	**2.92** (0.758)	0.**047**	15	2.76 (1.074)	15	3.36 (0.891)	0.107	0.193	0.157	0.879
Patient questions	15	**2.00** (0.740)	15	**2.72** (0.844)	**0.019**	11	**2.03** (1.007)	15	**3.08** (1.035)	**0.017**	0.916	0.306	0.489
CanMEDS health advocate	15	2.07 (0.594)	15	2.33 (0.724)	0.279	13	2.62 (1.044)	15	2.67 (0.976)	0.894	0.094	0.297	0.627

^*^Difference in development from t0 to t1 between groups. ^**^p-value for Satterthwaite's F-test. The bold values indicate significant at *p* < 0.05.

#### 3.5.3. Leadership

**Figure 6:**
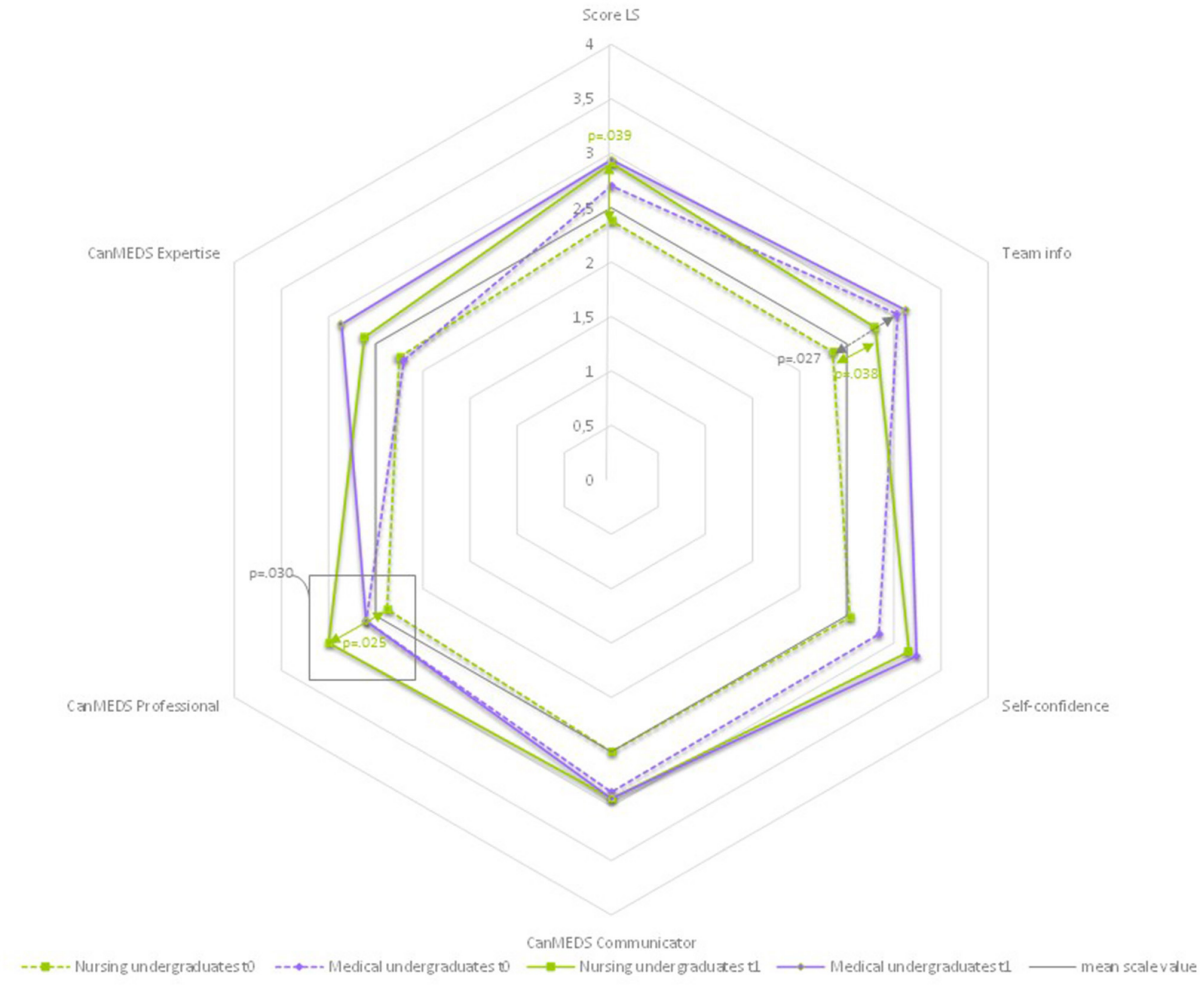
Differences by profession: leadership-spider chart of mean values.

For the subscale “*Leadership*”, as shown in Table 7 and Figure 6, no significant difference was found in the sum score between the two groups at either measurement time point. In t0, there was a significant difference in the mean values of both groups in the item “Ensures that all team members receive all information (team info)” (NU mean 2.35, MU mean 3.04, *p* = 0.027). At both t0 and t1, the mean values of the MU group showed a slightly higher value than the NU group in almost all items. However, all these differences are not significant. An exception is the item “CanMEDS Expertise”, where in t0 the mean value of the NU group was very slightly higher (NU mean 2.25, MU mean 2.20) than that of the MU group. This is exactly the other way around at t1 (NU mean 2.62, MU mean 2.86). Looking at both groups separately over time, the positive trend in the mean differences from t0 to t1 is highly significant for the MU group for this item (mean change 0.66, *p* = 0.003). This means that the medical undergraduates were more likely to be observed accessing and applying information to clinical practice and demonstrating diagnostic and therapeutic skills in t1 than in t0. In all other items, there was a positive trend in the MU group except for the item “CanMEDS Professional”, in which the mean value remained the same. On the other hand, the difference in the mean value for this item in the NU group is significant over time (mean change 0.62, *p* = 0.025). This means that the nursing undergraduates in t1 showed more often than in t0 behavior, which aimed to deliver high-quality care, and it was observed that they were involved in the ward round with professionalism and integrity. For the NU group, the differences of mean values t0 to t1 for the sum score (mean chance 0.53, *p* = 0.039) and the item “Team info” (mean change 0.45, *p* = 0.038) are also significant. The latter means that at t1, it was observed more frequently how the nursing undergraduates made sure, e.g., through eye contact or active inquiry, that the tandem partner took note of and understood the information that he or she had provided.

**Table 7:** Differences by profession – score “*Leadership*.”

	**t0**					**t1**					**Development**		
	**NU**		**MU**			**NU**		**MU**			**NU t0** > **t1**	**MU t0** > **t1**	**group diff** ^*^
	***n***	**Mean (SD)**	***n***	**Mean (SD)**	**p** ^**^	***n***	**Mean (SD)**	**n**	**Mean (SD)**	**p** ^**^	**p** ^**^	**p** ^**^	**p** ^**^
**Score LS**	16	2.38 (0.658)	15	2.7 (0.456)	0.120	15	2.91 (0.720)	15	2.94 (0.606)	0.896	**0.039**	0.231	0.336
Team info	16	**2.35** (0.805)	15	**3.04** (0.842)	**0.027**	14	2.80 (0.868)	15	3.12 (0.815)	0.738	**0.038**	0.739	0.141
Self-confidence	16	2.53 (0.979)	15	2.84 (0.767)	0.349	15	3.16 (0.901)	15	3.24 (0.920)	0.812	0.076	0.207	0.628
CanMEDS Communicator	16	2.50 (,730)	15	2.87 (0.516)	0.120	15	2.93 (0.884)	15	2.93 (0.799)	1.00	0.146	0.974	0.301
CanMEDS Professional	16	**2.38** (0.719)	15	2.60 (,507)	0.325	15	**3.00** (0.756)	15	2.60 (0.507)	0.100	**0.025**	1.00	**0.030**
CanMEDS expertise	12	2.25 (0.452)	15	**2.20** (0.561)	0.804	13	2.62 (0.506)	14	**2.86** (0.535)	0.240	0.071	**0.003**	0.308

^*^Difference in development from t0 to t1 between groups. ^**^p-value for Satterthwaite's F-test. The bold values indicate significant at *p* < 0.05.

**Figure 7:**
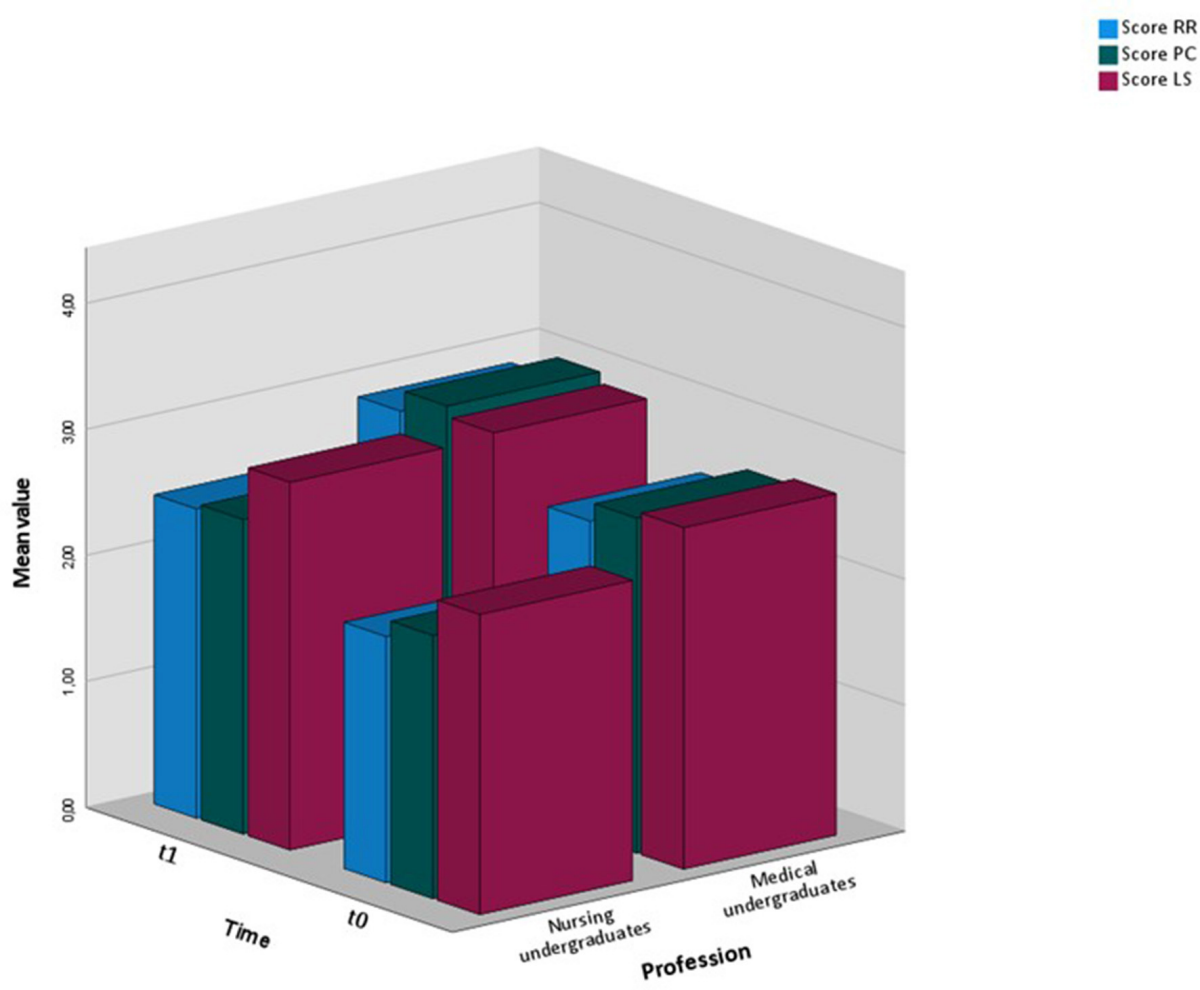
Bar chart of sum scores by group and time.

As can be seen in Figure 7, the maximum positive value (4.00) was not reached in any of the two groups at any time. The highest value in t1 (MU, Patient-Centeredness, mean 3.03) corresponds to 75.75% of the maximum value. The lowest value in t1 (NU, Roles and Responsibilities, mean 61.25) corresponds to 61.25% of the maximum value.

### 3.6. Relationship between individual competencies and team performance

We hypothesized (Hypothesis 4) that individual competencies and team performance are related. Pearson correlations were calculated to investigate whether there is a relation between individual competencies and team performance for all subscale sum scores.

As shown in Table 8, overall, within these four cohorts, at t0, there was only one significant moderate correlation for the individual and team score on “*Roles and Responsibilities*” (*r* = 0.416, *p* < 0.05). At t1, these scores showed a strong and highly significant correlation (*r* = 0.501, *p* < 0.005). The correlation between the individual and team scores on “*Patient-Centeredness*” was moderate and significant at t1 (*r* = 0.422, *p* < 0.05). Also at t1, there was a strong and highly significant correlation between both the individual scores on “*Roles and Responsibilities*” and “Patient-Centeredness” and the team score on “Decision-Making/CCR” (RR: *p* = 0.597, *p* < 0.001, PC: *r* = 0.516, *p* < 0.01). The non-significant negative correlation of the individual score on “Roles and Responsibilities” with the team score on “Patient-Centeredness” became a positive, although not significant, correlation at t1.

**Table 8:** Correlations of individual and team scores t0/t1.

	**t0 (*n =* 32)**			**t1 (*n =* 32)**		
	**Ind. RR**	**Ind. PC**	**Ind. LS**	**Ind. RR**	**Ind. PC**	**Ind. LS**
Team RR	**0.416** ^*^	0.041	0.306	**0.501** ^**^	0.225	0.002
Team PC	−0.096	0.254	0.033	0.238	**0.422** ^*^	0.251
Team CCR	0.153	−0.198	0.129	**0.597** ^**^	**0.516** ^**^	0.158^*^

RR, roles and responsibilities; PC, patient-centeredness; CCR, decision-making/CCR; LS, Leadership, ^*^p <0.05, ^**^p <0.01. The bold values indicate significant at *p* < 0.05.

Looking at group differences at t0 and t1 (Table 9), two significant (*p* < 0.05) strong correlations were found between the individual competencies of the MU group and team performance in t0, respectively, in the same categories “Roles and Responsibilities (RR)” (*r* = 0.567) and “Patient-Centeredness (PC)” (*r* = 0.562). Both correlations become even stronger at t1 (RR *r* = 0.715, PC *r* = 0.617) and highly significant (*p* < 0.01) in the former. For the NU group, the relation between individual and team scores for “*Roles and Responsibilities*” is also apparent but not significant. Also, not significant but noteworthy is the negative correlation in the NU group between all individual sum scores and the team score “*Patient-Centeredness*”. In t1, however, the correlations are positive, although not significant. Looking at the team scores for “*Decision-Making/Clinical Reasoning*” (CCR), it is noticeable that there are no significant correlations with the individual scores at t0, but that there are moderately to strong positive correlations with all individual scores in both groups at t1, which are significant or highly significant for the NU group (RR *r* = 0.767, *p* < 0.01, PC *r* = 0.611, *p* < 0.05, LS *r* = 0.633, *p* < 0.05).

**Table 9:** Correlations of individual and team scores t0/t1.

	**t0**						**t1**					
	**NU (*****n** =* **16)**			**MU (*****n** =* **15)**			**NU (*****n** =* **16)**			**MU (*****n** =* **15)**		
	**Ind. RR**	**Ind. PC**	**Ind. LS**	**Ind. RR**	**Ind. PC**	**Ind. LS**	**Ind. RR**	**Ind. PC**	**Ind. LS**	**Ind. RR**	**Ind. PC**	**Ind. LS**
Team RR	0.432	0.169	0.375	**0.567** ^*^	−0.004	0.319	0.416	0.094	−0.080	**0.715** ^**^	0.406	0.423
Team PC	−0.407	−0.130	−0.329	0.062	**0.562** ^*^	0.456	0.248	0.330	0.180	0.345	**0.617** ^*^	0.259
Team CCR	0.326	−0.189	0.105	−0.013	−0.239	0.192	**0.767** ^**^	**0.611** ^*^	**0.633** ^*^	0.446	0.472	0.502

RR, roles and responsibilities; PC, patient-centeredness; CCR, decision-making/CCR; LS, leadership, ^*^p <0.5, ^**^p <0.01. The bold values indicate significant at *p* < 0.05.

## 4. Discussion

### 4.1. Discussion of content

#### 4.1.1. Summary of key findings

For the evaluation of HIPSTA, structured ward round observation was conducted during the first and last weeks of the undergraduates' placement. In this subtask, conducting an interprofessional ward round, the NU and MU demonstrated significant competence acquisition in all three competence domains: “Roles and Responsibilities”, “Patient-Centeredness”, and “Leadership”. However, the two groups developed differently; while the NU mainly acquired competence in leadership and management, the MU developed professional expertise and was better able to define treatment goals and take over tasks at the end of their placement. Team performance also showed that roles and responsibilities were much more observable. It was striking that the mean values of the group of constant tandems at t1 were higher than those of the random tandems in all items except the “Swift effective rounds”. These differences were not significant. The development of individual competencies and team performance are related. It has been shown that this correlation increases over time.

#### 4.1.2. Integration into the body of research with external assessment on IPTW

Studies with a similar methodological approach are scarce, making it difficult to contextualize these results within the body of research. Brätz et al. (48) examined whether IPTW placement at the University Medical Center Hamburg-Eppendorf, Germany, had an impact on medical students' entrustable professional activities (EPA). After a 4-week placement in an IPTW (intervention group) or regular training (control group), 12 EPAs were recorded using a competency-based telemedicine assessment in a simulation of the first day of residency. The overall mean entrustment level was significantly higher (*p* < 0.001) in the IPTW group compared to the control group. Reeves et al. (32) and Freeth et al. (23) also conducted observations in the pilot of the Royal London Hospital's rheumatological and orthopaedical IPTW, UK. However, these were analyzed qualitatively and triangulated with data from individual and group interviews, so a systematic comparison of the results is not possible. Lidskog et al. (69) conducted unstructured observation when evaluating an IPTW within care for older people in Örebro, Sweden. In these studies, results were also triangulated with other qualitative data sources, so no comparison of the results of observation is possible. Same with the ethnographic observation conducted on an orthopedic ward at Karolinska Institutet, Stockholm, Sweden, by Ivarson et al. (70) that focused on a special learning intervention. The first result of the evaluation of the Mannheimer IPTW, Germany (37), identified ward round skills as a self-reported topic in which students gained competence.

#### 4.1.3. Discussion of findings concerning individual competencies and team performance

About the overall scale of individual competencies, it is interesting to note that the group-specific differences that existed at the beginning of the IPTW placement were only significant in two items at the end of the placement, which will be discussed in more detail. The groups did not develop differently, which may indicate that the educational concept of HIPSTA adequately supports both professional groups.

There was a statistically significant mean change in all subcategories of the individual competency scale, showing that participants seem to have improved their competencies, especially in terms of defining treatment goals, involving patients, and acting self-confidently. This confirms the results of the quantitative and qualitative (60) analysis of learners self-assessment of competency development and interprofessional socialization (61, 62) from an external, observational perspective. With regard to the development of the individual competencies subscale “*Roles and Responsibilities”*, the results described from questionnaire and interview studies (33, 49, 60, 62, 71–75) are substantiated and supplemented. The gain in understanding roles was also evident in the ward round observation. The participants behaved according to their professional roles and acknowledged the others' roles to a greater extent at the second team performance observation than at the first. Within the individual competency observation, an increase in self-confident/sovereign demeanor could also indicate a risen understanding of and identification with the professional identity. Higher confidence was also described in the interviews with participants 1–1.5 years after their placement on the HIPSTA (62).

##### 4.1.3.1. Patient-centeredness

With regard to the development of subscale “*Patient-Centeredness*”, no IPTW study so far has explicitly reported any effects. However, the Assessment of Interprofessional Collaboration Scale (AITCS) (76) and the Interprofessional Socialization and Valuing Scale (77, 78) were also used in the self-reported evaluation of HIPSTA (60). The AITCS, which includes aspects of patient-centeredness in the subscale “Coordination” showed a highly significant change in the mean sum score both in the pre-post as well as in the pre-follow-up comparison, and the ISVS, which covers patient-centeredness in terms of involving patients' interests and understanding and conducting collaborative decision-making together with patients, showed significant pre-post and pre-follow-up mean changes in the sum score and in the specific items (60). Within the analysis of learners retrospective evaluations of the HIPSTA, it has been shown that especially medical undergraduates had the impression of improved competencies in interprofessional communication in terms of listening to and understanding patient's needs (62). Still, these are all self-reported competencies, which do not guarantee performance. Hence, the results of this study give a better impression of how the undergraduates actually demonstrate their self-perceived competencies. The patient is central to the frameworks for interprofessional collaboration and the starting point for the call for more ICPC and IPE (1, 6, 38, 39). Analyses of the concept of “patient-centeredness” show that it is rich in perspectives and dimensions and requires further research to be operationalized for the health professions (79–82). Spaulding et al. (83) identify a lack of research on the patient-centeredness outcome of IPE. Orchard (84) sees the nursing leader's role as key between patients and other health providers. Interestingly, no significant differences in the patient-centeredness items from t0 to t1 were found in the NU group, and the sum score in the subscale “Patient-Centeredness” was significantly lower in the group of NU compared to the group of MU sum scores at both points in time. This could be related to the way in which patient-centeredness was recorded in the ward rounds, namely primarily with the extent to which the patient was involved in obtaining information and the extent to which questions were motivated and answered. Also, since ward rounds serve to clarify the patient's medical condition, it is not that surprising that there was a certain patient-centeredness present and observable. This explains the difference in “ensures that the patients' questions are asked and answered”, which is also significant at t1. Furthermore, there was a given structure for the ward round that was co-developed by the learners, saying that in the first step, the nursing undergraduate introduces the patient and reports on the process; second, the medical undergraduate takes the lead of the round; third, the patient is asked an open question (“how are you”); and fourth, a joint evaluation of the situation and background takes place. Having the lead of the round could have made the medical undergraduates feel more responsible for patient involvement than the nursing undergraduates in this specific situation. In addition to that, most nursing undergraduates have already visited the patients' rooms and talked to them in the morning before the ward round. For them, it might have been artificial to have the same conversation a second time. Since the undergraduates followed a structure, medical undergraduates' improvement in patient-centeredness does not necessarily mean that they actually change their attitude toward the patient; it could also indicate that they were better able to implement the instructions on the round. Within the team performance scale, no statistically significant improvement in the sum score “*Patient-Centeredness*” was shown. Still, the mean score of patient involvement in information collection was rather positive at t1.

##### 4.1.3.2. Individual development of nursing and medical undergraduates

Regarding the subscale “*Leadership*”, the nursing undergraduates underwent a significant change, although the item “CanMEDS professional” was the only item where the mean change of both groups differed significantly. Also, at t1, the nursing undergraduates paid more attention to ensuring that all team members received all relevant information and backed this up. They also took on more of a managerial role (CanMEDS Manager) compared to that in t0. If this is viewed in parallel with the development of medical undergraduates, who developed especially in terms of goal definition and the assumption of tasks, professional socialization can be surmised. Wenger (85) described in the “communities of practice”, which was later described as a concept in health education (86), that professional identity forms in dependency on the relations and activities of other members of the community. This can also be seen in the statistically significant improvement of values regarding role assignment within the team performance scale. From this perspective, the interprofessional setting of the ward round could be conducive to the professional identity of medical and nursing undergraduates. This has also been shown by the longitudinal quantitative analysis of the HIPSTA within the ISVS that also measures role clarification (60) and within follow-up interviews with the participants, where they described an improvement in confidence and self-efficacy in their professional role due to the experience on the HIPSTA (62). In this study, it has been observed that medical students acquired and applied skills in collaborative clinical reasoning and decision-making, whereas nursing undergraduates acquired leadership skills. The importance of collaborative clinical reasoning skills in medical education has been widely acknowledged (87). Leadership skills for nursing are key for ward management and team performance in healthcare. It might have a central role in education and should be further investigated. In her review, Cummings et al. (88) analyzed factors and educational interventions that influence nursing leadership. However, they were unable to include any studies in an interprofessional setting. Orchard et al. (89) advocate nursing leadership as a dual role in interprofessional teams, namely, managerial and disciplinary. They suggest that in areas nurse leaders are responsible for, “their ability to support health providers use of knowledge, skills, and expertise to address the complex and uncertain needs of those persons seeking help can result in improved care”.

The concept of professional identity was further developed by Khalili et al. (90, 91) for the interprofessional context, forming the concept of dual identity and professional socialization (interprofessional socialization framework). Thistlethwaite (92), referring to Miller's competence pyramid (93), describes it similarly by asking if it needs a fifth competency level “is” above “does”. Mink et al. (61), referring to the concept of dual identities, examine in a reconstructive analysis of the focus groups of the first cohorts of HIPSTA the extent to which interprofessional socialization has occurred and conclude that it cannot be reliably anticipated. The data of cohorts 8–11 examined here show, in comparison to the constant with random tandems, that the former tended to plan the further procedure together significantly more often than the random tandems. The mean score for the constant tandem at t1 is above the middle, resembling a positive evaluation of their collaborative planning. However, no statement can be made about the participants' sense of belonging to the interprofessional community or about how sustainably team performance can be implemented in the subsequent everyday work.

The problem, also in evaluating interprofessional teaching by means of patient-relevant outcomes, is that little insight is gained into the black box between IPE and IPCP. IPE is important and has a positive impact on attitudes and competencies. Good IPCP increases patient safety and quality of care. But how does the former relate to the latter? One approach could be to break down the huge field of IPCP into small bites by asking what clinical problem is specifically to be solved by better interprofessional collaboration. This study is based on the premise that interprofessional competencies can be observed particularly well in interprofessional rounds. The ward round could therefore be seen as a clinical problem, as a unit of care structure in the clinical setting in need of optimization, which should be solved or optimized through IPCP. About half of all adverse events in the surgical setting occur outside the operating room (94) and are associated with poor organization of inpatient care. The ward round is a central element of quality assurance because it is used to exchange information, record the patient's condition, and plan further procedures within the team, if things are going well. If things are not going well, this can have a correspondingly negative effect on patient care. Klaas et al. (95) propose a taxonomy of non-technical skills for the surgical ward round and define good and bad behavior for the team and the team leader in four categories, namely, “Leadership”, “Situation awareness”, “Decision-making”, and “Communication and teamwork”, which was evaluated for nurses (96), and which are complemented by our study results very well. In this respect, a very small crack in the black box is opened in that the IPTW setting enhances a concrete clinical activity, ward rounds.

### 4.2. Discussion of methods and limitations

The study was single-centered and was conducted without a control group. The sample is small, which limits the statistical possibilities, and due to this, it should be considered that statistical tests have low power with small effect sizes. Therefore, both “almost” significant and non-significant changes have been reported and discussed.

For the interpretation of the results of this study, differences in the group comparisons between nursing and medical undergraduates might not (only) result from the professional background but from age or gender, or at least co-variances exist, which could not be examined in more detail due to the small sample size. In Germany, the medical study program takes twice as long as nursing school, and nursing is still predominantly female.

The data presented here were collected using the previous version of the IP-VITA (IP-VITA^pre^), which has so far only been validated descriptively (63, 97) and not statistically. There were a few adaptations after the observations in the four cohorts, the results of which are presented here. The CanMEDS roles in IP-VITA^pre^ were adopted as items without critically appreciating previous publications on the recording of CanMEDS and its complexity (98–104). Although this framework was initially physician-specific, it has been successfully transferred to other health professions (105–108). However, the researchers did not operationalize further into the items but instead referred to the role model in interpretive intersubjective exchange to assess the undergraduate's behavior. This worked well for the observations in the HIPSTA setting. However, for a transfer to other sites and the use of the instrument, possibly with only one observer, more extensive descriptors and a further operationalization of the roles would have been necessary. As a consequence, the CanMEDS roles were removed. The two individual items “Discusses current patient information with patient involvement” and “Ensures that the patients' questions are asked and answered,” were split into five more distinct items (see IP-VITA in the Supplementary material). Adjustments were also made to the team performance scale, aimed at a clearer delineation of the items. In addition, it was also scaled to six-point instead of four-point Likert, and a distinction was made between observable interaction during the ward round and during debriefing.

The ward round observations were conducted by three researchers (AMit, CA, and JM) in different constellations of two. The observers had nursing (*n* = 2) and gerontological (*n* = 1) backgrounds, each with academic degrees. None had a medical background, and none of the three had specific training as observers; nonetheless, all had experience in quantitative and qualitative research. Because assessment practices are by no means trivial (109), assessment literacy (110) may be questioned, at least with respect to items related to medicine (e.g., physician decision-making and goal setting). The way the researchers handled this was to involve the physician learning facilitators when there was uncertainty.

For the interpretation of the data, especially the changes from t0 to t1, it is important to point out again that the group of tandems was naturally half as large as the group of individuals. The fact that there were more significant changes in the mean values of the individual scale from t0 to t1 could be due to the fact that the tandem sample is smaller than the individual one.

IPTWs are highly complex learning interventions. This complexity is highly conducive to the cause of IPL/IPE late in vocational training and study (111) – but not to its research. The setting of the observations that produced the data presented varied in terms of the number of patients, others (passively) involved, and the participants themselves to be observed. The setting was not meticulously recorded for each observation in this study. For exploration in the pre-post design, it would be necessary for the tandems to be composed of the same individuals at t1 as at t0. This was only the case to a very small extent in this study. The effects of the different individuals in the tandems were accounted for in the linear mixed model, but this still limits the interpretation.

For further studies, it would be advisable to reduce some of the complexity of the setting and to standardize the framework conditions as far as possible. Since patients cannot be standardized with regard to their illnesses and real-patient contact is the special attraction of observing interprofessional interaction, the other parameters should be adjusted. First and foremost, the tandems or teams from which data are collected should be identical at the time of collection. On the other hand, perhaps the very fact that they are not is the right approach. If the premise were that individual competencies in any healthcare team should have a positive impact on quality of care, then this might be an interesting idea to think about further, at least for formative feedback. What could be standardized for a follow-up survey would be that the individuals who participate in the ward round are defined, and clear guidelines also apply regarding their contribution. Furthermore, the course of the round could be standardized insofar as it was often not clear in this study when exactly the visit and thus the observation began and when it ended. Our study collected data on a small but important part of the IPTW, the ward round. However, multi-center approaches should also have the learning process of the IPTW itself in focus. Further studies could observe other team tasks on IPTW, like handovers and collaborative clinical reasoning. Also, longitudinal studies with repeated data collection and analysis several weeks and months after an IPTW placement should be conducted.

## 5. Conclusion

This study describes how interprofessional tandems at the end of an IPTW assignment interact more clearly in terms of their roles and tasks, are more patient-focused, and are better able to obtain and share information to set goals for treatment and plan next steps as a team. Our evidence suggests that tandems that stay consistently together perform better than tandems with changing partners. If this finding manifests itself, IPTW could be organized so that learning teams should be stable and not change. Alternately, IPTW research could focus on developing learning support approaches with prompts and intermediate learning goals that allow medical and nursing post-graduates to bring interprofessional competencies to performance independent of the tandem partner or team. We consider the latter to be the more promising way to foster the transferability of individual competencies to later team performance. In a work environment, healthcare teams change quite regularly. Therefore, the aim of IPTW should be to prepare healthcare team members for this change. Further studies will also focus on the translation of learned interprofessional competence into later professional practice. Perhaps IPTW, with its externally valid approach and high complexity, are one of the messiest research settings in healthcare education. Because of their high cost and organizational effort, it is our duty as healthcare education researchers to design IPTW for the best learning environment possible. Aside from team stability, there is much to be found out.

## Data availability statement

The raw data supporting the conclusions of this article will be made available by the authors, without undue reservation.

## Ethics statement

The studies involving humans were approved by the Ethics Committee of the Medical Faculty Heidelberg (S-072/2017). The studies were conducted in accordance with the local legislation and institutional requirements. The participants provided their written informed consent to participate in this study.

## Author contributions

AMit, JM, and CM conducted the study, acquired financial support, and developed the design of methodology. AMit, JM, and CA collected data, conducted the research, and investigation process. BT-H and AMih provided resources that enabled the study to be conducted. AMit conceptualized this work, led the analysis, interpretation, and wrote the initial draft of this manuscript. AMit and AMö performed the statistical analysis. CU supervised the development of the IP-VITA instrument and the qualitative aspects of the observations. JK supervised the work. All authors corrected and approved the revisions and final version of the manuscript.
