# Supplementary material for: Development of individual competencies and team performance in interprofessional ward rounds: results of a study with multimodal observations at the Heidelberg Interprofessional Training Ward
Source: Front Med (Lausanne). 2023 Sep 27;10:1241557. doi: 10.3389/fmed.2023.1241557 (PMC10566636; doi:10.3389/fmed.2023.1241557)
Supplement: Supplementary file 1 [file Data_Sheet_1.PDF]

UniversitätsKlinikum Heidelberg

IP-VITA

Interprofessional Ward Rounds Individual and Team Assessment-Tool

Mitzkat A[a*], Mink J[a], Arnold C[a], Krug K[a], Mahler C[b], Trierweiler-Hauke B[c], Wensing M[a], Kiesewetter J[d], Mihaljevic AL[e], Ullrich C[a]

Part A: Setting of the Ward Round

Date: __ __/__ __/ 20____ Start: ______ End: ______

Cohort: _________ ☐ T0 ☐ T1

Nurs. Facilitator: ______________________ Med. Facilitator: _______________________________

Other Participants: __________________________________

____

____

Observer 1: _______________ NU 1: ______________

NU 2: ______________

Observer 2: _______________ MU 1: ______________

MU2 : ______________

Specifics: ______________________________________

	Diagnosis and Specifics	Room	Team	Individual
**Patient 1**		1	Team _____ , Tandem 1<br>NU Pseudonym: __________<br>MU Pseudonym: __________	No.:<br>No.:
**Patient 2**				
**Patient 3**				
**Patient 4**		2	Team _____, Tandem 2<br>NU Pseudonym: __________<br>MU Pseudonym:__________	No.:<br>No.:
**Patient 5**				
**Patient 6**				

[a] Abteilung Allgemeinmedizin und Versorgungsforschung, Universitätsklinikum Heidelberg, Heidelberg, Deutschland
[b] Abteilung Pflegewissenschaft, Universitätsklinikum Tübingen, Tübingen, Deutschland
[c] Klinik für Allgemein- Viszeral- und Transplantationschirurgie, Universitätsklinikum Heidelberg, Heidelberg, Deutschland
[d] Institut für Didaktik und Ausbildungsforschung in der Medizin, Universitätsklinikum München, München, Deutschland
[e] Klinik für Allgemein- und Viszeralchirurgie, Universitätsklinikum Ulm, Ulm, Deutschland

Pseudonym: [ ] Individual Observation No. [ ] ☐T0 ☐T1

To be completed by one observer per MU or NU during the observation

Part B: Individual Competencies

**During the ward round**	to very high degree					to a very low degree	not observable
Discusses current patient information	☐	☐	☐	☐	☐	☐	☐
Involves the patient to receive information	☐	☐	☐	☐	☐	☐	☐
Gives the patient opportunities to ask questions	☐	☐	☐	☐	☐	☐	☐
Responds to patients' questions	☐	☐	☐	☐	☐	☐	☐
Ensures that the patient is adequately informed about further procedure	☐	☐	☐	☐	☐	☐	☐
Ensures that all team members receive all information	☐	☐	☐	☐	☐	☐	☐
Defines clear goals for further treatment	☐	☐	☐	☐	☐	☐	☐
Takes over tasks	☐	☐	☐	☐	☐	☐	☐
Distributes tasks	☐	☐	☐	☐	☐	☐	☐
Self-confident/sovereign appearance	☐	☐	☐	☐	☐	☐	☐
Recognizes own knowledge gaps and asks questions	☐	☐	☐	☐	☐	☐	☐
Active participation	☐	☐	☐	☐	☐	☐	☐

Pseudonym: [ ] Individual Observation No. [ ] ☐T0 ☐T1

To be completed by one observer per MU or NU during the observation

Part B: Individual Competencies

**During the ward round**	to very high degree					to a very low degree	not observable
Discusses current patient information	☐	☐	☐	☐	☐	☐	☐
Involves the patient to receive information	☐	☐	☐	☐	☐	☐	☐
Gives the patient opportunities to ask questions	☐	☐	☐	☐	☐	☐	☐
Responds to patients' questions	☐	☐	☐	☐	☐	☐	☐
Ensures that the patient is adequately informed about further procedure	☐	☐	☐	☐	☐	☐	☐
Ensures that all team members receive all information	☐	☐	☐	☐	☐	☐	☐
Defines clear goals for further treatment	☐	☐	☐	☐	☐	☐	☐
Takes over tasks	☐	☐	☐	☐	☐	☐	☐
Distributes tasks	☐	☐	☐	☐	☐	☐	☐
Self-confident/sovereign appearance	☐	☐	☐	☐	☐	☐	☐
Recognizes own knowledge gaps and asks questions	☐	☐	☐	☐	☐	☐	☐
Active participation	☐	☐	☐	☐	☐	☐	☐

Tandem: NU: MU: ☐ T0 ☐ T

To be completed by one observer during each visit

Observer

Part C: Team Performance

Before the ward round		
Tandem 1	Room 1	
	Patient 1	
	Patient 2	
	Patient 3	
Tandem 2	Room 2	
	Patient 4	
	Patient 5	
	Patient 6	
Debriefing after the ward round		

Tandem: [ ] NU: [ ] MU: [ ] ☐ T0 ☐ T

To be completed by one observer during each visit

Part C: Team Performance

**During the ward round**	to very high degree					to a very low degree	not observable
Exchange between NU and MU present	☐	☐	☐	☐	☐	☐	☐
Relevant nursing information present	☐	☐	☐	☐	☐	☐	☐
Relevant medical information present	☐	☐	☐	☐	☐	☐	☐
Patient is involved in information collection	☐	☐	☐	☐	☐	☐	☐
Patient is involved in decision making process	☐	☐	☐	☐	☐	☐	☐
Patient asks questions	☐	☐	☐	☐	☐	☐	☐
Patients' questions are answered	☐	☐	☐	☐	☐	☐	☐
Goals are defined with the patient	☐	☐	☐	☐	☐	☐	☐
Further procedure is planned by the team	☐	☐	☐	☐	☐	☐	☐
Responsibilities are clarified	☐	☐	☐	☐	☐	☐	☐
	☐	☐	☐	☐	☐	☐	☐
Structured procedure of the round	☐	☐	☐	☐	☐	☐	☐

**After the ward round**	to very high degree					to a very low degree	not observable
Exchange between NU and MU present	☐	☐	☐	☐	☐	☐	☐
Relevant nursing information present	☐	☐	☐	☐	☐	☐	☐
Relevant medical information present	☐	☐	☐	☐	☐	☐	☐
Patients' perspective is involved in decision making process	☐	☐	☐	☐	☐	☐	☐
Patients' perspective is involved in setting goals	☐	☐	☐	☐	☐	☐	☐
Further procedure is planned by the team	☐	☐	☐	☐	☐	☐	☐
Responsibilities are clarified	☐	☐	☐	☐	☐	☐	☐
Roles are clearly assigned	☐	☐	☐	☐	☐	☐	☐

SUPPLEMENT: Clarification of terms and descriptors of the items

Part A: Setting of the ward round

Team: one shift (early/late) on the ward = 2 nursing and 2 medical undergraduates

Tandem: one nursing and one medical undergraduate responsible for one patient room

MU: medical undergraduate

NU: nursing undergraduate

Nurs./Med. Facilitator: Nursing/Medical Learning Facilitator

Observer: ID of the observing person

Specifics concerning the setting: deviations from regular procedure that could influence the ward round (e.g. chief physician ward round)

Specifics concerning the patient: e.g. panned examinations and consults

Individual observation number: see header of part B

Part B: Individual Competencies

Discusses current patient information: NU or MU exchanges information with the tandem partner in a structured way. A dialogue takes place in which the information is merely passed on but backed up collegially

Patient is involved in information collection: NU or MU actively and clearly addresses the patient to obtain or verify information

Gives the patient opportunities to ask questions*:* NU or MU makes sure that the patient has time to ask questions and actively askes for it

Responds to patients' questions: NU or MU responds transparently and in a language that the patient can understand. If answering the patients' question is not possible, she/he provides information about when she/he will discuss the question later with the patient

SUPPLEMENT: Clarification of terms and descriptors of the items

Ensures that the patient is adequately informed about further procedure: NU or MU ask the patient questions to find out about his/her level of knowledge and/or has the patient repeat the agreements made

Ensures that all team members receive all information: NU or MU ensures that the tandem partner or team member have taken note of and understood the information by making eye contact and/or actively asking

Defines clear goals for further treatment: NU or MU prioritizes the further course of treatment and specifies feasible goals including their implementation in terms of time

Takes over tasks: NU or MU actively expresses what he/she will do next

Distributes tasks: NU or MU actively askes his/her tandem partner to complete specific tasks during the rest of the day. In doing so, she/he also exchanges information about the time of competition

Self-confident/sovereign appearance: NU or MU is articulate an gives the impression of being confident about the process and tasks in the ward round

Recognizes own knowledge gaps and asks questions: Nu or MU asks questions of understanding or asks his/her tandem partner or the learning facilitators for guidance in an action in which he/she feels uncertain

Active participation: NU or MU uses information and/or questions to be clarified proactively in the ward round and is merely reactive.

Partl C: Team Performance

Exchange between NU and MU present: NU and MU converse during/after the ward round about relevant aspects of their patients' diagnosis/care/treatment

Relevant nursing information present: All team members know all relevant aspects concerning nursing care of the patient

Relevant medical information present: All team members know all relevant aspects concerning medical treatment of the patient

Patient is involved in information collection: The patient adds information relevant to him/her or corrects it as needed

SUPPLEMENT: Clarification of terms and descriptors of the items

Patient is involved in goal setting: The patient actively participates in planning further treatment and contributes his/her wishes/needs and suggestions

Patient asks questions: The patient asks questions if anything is unclear to him/her

Patients questions are answered: NU and MU answer all the patient's questions. If they can not answer the question right now they offer to do so at a concrete later time

Goals are defined with the patient: All team members including the patient formulate goals that are discussed and set together

Further procedure is planned by the team: All team members including the patient contribute their opinion and discuss together the further treatment of the patient

Responsibilities are clarified: NU and MU show by their statements and behavior that they know what their profession-specific tasks are in the area of diagnostics/care/treatment of the patient and what the tasks of the other professional groups are

Rolls are clearly assigned: NU and MU behave according to their professional role and recognize the role of the other.

Structured procedure of the ward round: The ward round is structured in an effective way so that all aspects are presented in a sensible sequence
